# User Experiences of Pharmacogenomic Testing and Opinions among Psychiatry Patients

**DOI:** 10.3390/jpm14010022

**Published:** 2023-12-23

**Authors:** Catherine R. Virelli, Mahbod Ebrahimi, Ayeshah G. Mohiuddin, Julia Tomasi, Amanda J. Lisoway, Deanna Herbert, Victoria S. Marshe, Sean A. Kidd, Joseph Ferenbok, James L. Kennedy

**Affiliations:** 1 Tanenbaum Centre for Pharmacogenetics, Campbell Family Mental Health Research Institute, Centre for Addiction and Mental Health, Toronto, ON M6J 1H4, Canadamahbod.ebrahimi@mail.utoronto.ca (M.E.); julia.tomasi@mail.utoronto.ca (J.T.);; 2 Translational Research Program, University of Toronto, Toronto, ON M5S 1A8, Canada; 3 Institute of Medical Science, University of Toronto, Toronto, ON M5S 1A8, Canada; 4 Department of Neurology, Columbia University, New York, NY 10027, USA; 5 Department of Psychiatry, University of Toronto, Toronto, ON M5S 1A8, Canada; 6 Laboratory Medicine and Pathobiology, University of Toronto, Toronto, ON M5S 1A8, Canada

**Keywords:** pharmacogenomics, pharmacogenetics, mental health, personalized medicine, focus group

## Abstract

Pharmacogenomic testing (PGx) is a tool used to guide physicians in selecting an optimal medication for clients based on their genetic profile. The objective of this qualitative study is to understand patients’ experiences with PGx testing as well as their opinions regarding the clinical adoption of such tests in psychiatry. A focus group was conducted to assess the needs of clients who had experience using a PGx test. Participants were recruited from a large study on PGx testing that offered physicians an opportunity to use PGx reports to guide psychotropic prescriptions. The focus group discussions were recorded, transcribed, and coded using NVivo to identify core themes. A total of 11 people participated in the focus group. Our analysis revealed that many participants were in favour of implementing PGx testing in psychiatric practice, and all expressed important considerations for patient-centred optimization of PGx testing. The main themes captured were: education and awareness among clinicians, cost considerations, PGx results-sharing and accessibility, and prospective benefits. The results of this study suggest that patients are keen to see PGx testing in widespread clinical care, but they report important opportunities to improve knowledge mobilization of PGx testing.

## 1. Introduction

Pharmacogenomic testing (PGx) is a tool used to guide physicians in selecting an optimal medication for clients based on their genetic profile. Pharmacological approaches, including antidepressants and antipsychotics, are a mainstay treatment for psychiatric disorders. Such medications are mostly chosen for a patient on the basis of trial and error. This approach involves selecting one or more of the most commonly prescribed medications for a given condition, assessing the client’s response, and making corresponding dosage adjustments or changing medications altogether. The trial-and-error approach to medication prescription can have problematic treatment outcomes for patients experiencing mental illness, given that it can take several weeks (typically 4–6 weeks) for an effect to be seen, and adverse side effects can occur that affect patients’ subjective sense of well-being and ability to function in day-to-day life [1]. Notably, fewer than 40% of patients with depression achieve remission during their first medication trial, with rates declining in additional trials [2]. As a result of this lengthy process and lack of treatment success, patients lose hope in their healthcare system, and many do not adhere to their prescribed treatment regimen, which can impair their recovery [3,4]. Thus, there is an urgent need for personalized treatment selection to remedy the prominent downfalls of trial-and-error approaches to psychiatric medication prescription. Moreover, a meta-analysis of prospective controlled clinical trials has revealed that people with PGx-guided therapy could achieve 40% higher remission rates than unguided therapy, illustrating the significant effects of PGx-guided medication selection [5].

Medication metabolism is one of the important factors that affect the way in which a client responds to medication and is well known to vary between individuals largely as a result of their unique genetic makeup [6,7,8]. The field of pharmacogenomics explores interactions between medications and genetic factors that play a role in medication metabolism, such that prescriptions can be tailored to an individual based on their genetic profile. Genetic variants in the liver enzyme genes *CYP2D6*, *CYP3A4*, *CYP1A2*, *CYP2C19*, and *CYP2C9*, as well as the serotonergic pathway genes *SLC6A4* and *HTR2A*, play an important role in psychotropic medication metabolism [9,10,11]. Thus, PGx testing, including these genes, is promising in predicting clinical efficacy, improving adherence, and reducing healthcare costs, as shown in several studies [12,13,14].

PGx testing has only had limited adoption in clinical settings, including psychiatry. Several studies and regulatory bodies have identified a number of barriers preventing this clinical adoption in international contexts. For example, the lack of physician knowledge regarding PGx, the generalizability of clinical utility for PGx testing, and the cost-effectiveness of PGx have been among the factors impeding the widespread implementation of PGx testing [15,16,17,18,19,20,21,22,23]. In addition to these barriers, the literature is noticeably lacking reports investigating patient needs related to the clinical adoption of PGx testing. While the number of studies that have directly solicited physicians and patients has improved in the past several years [24,25,26,27,28], few investigate patient needs for PGx testing in the context of psychiatric care, specifically [29].

To address the gap in the literature soliciting public and user opinions and perspectives regarding PGx testing, a focus group study was conducted with participants from the Individualized Medicine Assessment and Clinical Testing (IMPACT) study. The IMPACT study, developed at the Centre for Addiction and Mental Health (CAMH), was a seven-year province-wide naturalistic research study to test the feasibility and implementation of PGx testing for psychiatric medications in Ontario, Canada. The IMPACT study offered the GeneSight Psychotropic PGx report (Myriad Genetics^TM^, Zurich, Switzerland) to the treating physicians of individuals experiencing mental illness [29]. The objective of the present study was to learn about participants’ experiences using the PGx test, the ways in which the test influenced their treatment, and their needs and opinions as related to the clinical adoption of PGx testing in psychiatric contexts.

## 2. Materials and Methods

### 2.1. Study Participants

Eligibility for participation in the focus group was restricted to participants from the CAMH IMPACT study who (i) had consented to be re-contacted for future studies, (ii) had completed all follow-ups from the IMPACT study, and (iii) had participated in the study in the years 2017 and 2018. This criterion was set to improve the likelihood that participants could accurately recall and reflect on their experience. All other inclusion criteria matched that of the IMPACT study [29]. An invitation email was sent to those who matched the inclusion criteria. The email also included a link to registration that maintained participant anonymity. Interested participants completed the survey, which did not require participants to disclose personally identifying information, and were sent information about the time, date, and location of the focus group.

### 2.2. Facilitators

Facilitators were staff and students from the Molecular Brain Science department at CAMH who had received a workshop session of informal training in group facilitation relevant to the approved protocol, informed by published guidelines from the NSW Agency for Clinical Innovation [30]. The training included avoiding biasing participants’ comments, maintaining appropriate privacy of personalized information, moderating group discussions, and balancing contribution time across participants.

### 2.3. Focus Group Guide Design

The focus group guide included eight questions (Table 1). The first three questions solicited feedback related directly to participants’ experience in the IMPACT study, including benefits and concerns related to PGx testing. The following questions ask participants to consider key aspects related to the clinical adoption of testing—i.e., cost of tests, distribution, etc.—and provide their opinion regarding best practices based on their own experience using the test. These questions were developed based on literature regarding key barriers to the adoption of PGx testing. Questions were not provided to participants in advance of the focus group. The focus group event took place in October 2019. See Appendix A for a detailed explanation of each question.

### 2.4. Procedure

Facilitators asked questions from the guide in sequential order. In order to stay within the time scheduled for the focus group, facilitators were asked to allow up to 10 min for discussion of each item. However, facilitators were advised to allow all participants the opportunity to contribute to each question and to avoid interrupting the conversation. All discussions were recorded in order to maintain the quality and accuracy of data transcription.

To understand whether the current market price of PGx testing was accessible to participants, participants were invited to share anonymously the price they would be willing to pay, out-of-pocket, for the PGx test they used (see Question 4). Participants indicated the price on a slip of paper and were asked to pass their responses to the facilitator at the end of the focus group. Facilitators deposited all slips of paper in a single envelope at the end of the event.

### 2.5. Data Analysis

Focus group recordings were transcribed manually and coded independently by two separate investigators using NVivo. First-pass coding identified ideas and sentiments expressed in each focus group discussion; second-pass coding grouped these codes into broader themes common across focus groups. Coding was conducted independently by the first and second authors, and codes were compared to identify common core themes. Here, we report the most commonly coded themes of the data set.

## 3. Results

A total of 30 IMPACT participants accepted the email invitation to the focus group. Of these, 19 participants did not show up, and 11 attended the focus group event and participated in the focus groups. The mean age of participants was 45.36 years; approximately 82% of the sample self-reported European ancestry, and 18% of the sample reported East Asian Ancestry. Table 2 shows the specific age categories of participants. Upon arrival at the event venue, each participant was placed into one of three groups, resulting in two groups of four and one group of three participants. Two facilitators were assigned to two of the groups, and one facilitator to the remaining group.

Final coding resulted in six (6) key themes: barriers and unmet needs, cost considerations, PGx results-sharing and accessibility, clinical utility, prospective benefits, and IMPACT study experience. Some of these broad themes contained more specific sub-themes, including the need for improved education and room for improved results layout (under the barriers and unmet needs core theme).

### 3.1. Barriers and Unmet Needs

The Barriers and Unmet Needs theme included codes describing barriers to participants’ ability to access, effectively use, and/or benefit to their desired extent from PGx testing. It also included the needs associated with these barriers. Since the coded needs address one or more barriers coded within this theme, here, we share the two most commonly coded needs.

#### 3.1.1. Need for Improved Education and Knowledge Mobilization for Health Service Providers

Lack of physician education regarding PGx testing was highlighted by participants as a concern in accessing and potentially benefiting from tests. Of all focus group participants, only two reported that their physician had sufficient knowledge to discuss test results with them in satisfactory detail. Among the participants whose physicians appeared to lack knowledge regarding PGx testing, experiences varied concerning physicians’ willingness to use test results to guide prescription selection. One participant reported their physician did not feel comfortable proceeding with a recommendation based on test results.

Participants attributed the inability of their health service provider to meet their knowledge needs to an overall lack of formal education regarding PGx among physicians and an absence of supplemental, descriptive information regarding PGx and test interpretation in the results report. Consequently, participants expressed a need for their clinicians to have basic and accessible PGx information, as well as guidance for test use.

*“It would be nice to have a one-on-one with somebody who’s a little deeper into the testing and the research that could say ‘hey, this is where we’re at, this is what we found out about you”*

While it was suggested that better education for PGx is required, the test results were sufficient to point out drugs that potentially would work for each participant. This knowledge of individualized test results aided participants in having a clearer view of medications that would benefit them.

Participants across all three focus groups described multiple positive attributes of the testing; however, all three expressed that improved physician education and awareness regarding PGx testing would be very helpful for clinical adoption.

#### 3.1.2. Need for Education and Effective Knowledge Translation Regarding PGx among Test-Users, and Members of the Public

Many participants expressed a need for basic education and corresponding information regarding PGx testing to be made available to the general public, as well as patients. Increased availability of informational and educational resources related to PGx testing and the role of genetics in medication metabolism, generally, was commonly considered important. Two participants cited this as the most important consideration for clinical adoption (see Question 8, Table 1). Information specific to PGx testing that participants commonly considered important included a plain-language description of how results are produced and a summary of what they mean for patients’ care. Participants in the two groups expressed that such an explanation would be especially important to maintain positive morale among users who had few to no medications in the ‘Use as Directed’ category and would also be important to manage users’ expectations of the extent to which test results could inform their treatment.

*“I think if this [PGx testing] were made available to patients, one of the things that would be required would be like, an information sheet in terms of how this information is useful.”*

Many participants also emphasized the importance of building awareness and mobilizing information about PGx testing and personalized medicine for general public audiences. This would further enable patient empowerment. In two groups, participants discussed the potential of PGx testing to counter the stigma associated with taking psychotropic medications.

*“If something can be tested, then, you know, it’s a molecular thing. Like, it’s not mental, it kinda destigmatizes mental illness to a certain degree.”*

*“I didn’t really learn any of this in school, but like…it’s more symptoms [of mental illness] that I feel like are being taught in school, but not really the science behind it. And I know that there’s both….It’s a lot easier for people to understand in order to destigmatize mental health.”*

### 3.2. Cost Considerations

Market prices for PGx tests are influenced by a number of factors, including—but not limited to—distribution channels (e.g., direct-to-consumer vs. physician-administered) as well as the extent of research and development invested in each test. Given that test costs would inevitably influence the feasibility of clinical adoption, participants were asked to anonymously share the out-of-pocket price that, at the time of discussion, would be feasible for them to pay for a PGx test. The mean cost participants were willing to pay for testing was $828 CAD (627 USD in October 2019). Factors that influenced willingness to pay included personal income and other financial supports, financial independence, education of the scientific and healthcare communities regarding PGx, and availability of empirical evidence supporting the clinical efficacy of PGx testing.

In Canada, clinical adoption of PGx testing and access to PGx testing by the public would be considerably facilitated by coverage under provincial health insurance plans. Participants were asked whether, based on their experience with the test, they would be willing to support government subsidization, given the potential for a consequent increase in personal income tax. Eighty-two percent of participants (*n* = 9) expressed support for public funding of tests, even with associated tax increases—many of these participants believed that PGx tests have the potential to reduce the number of medications prescribed to individual patients and facilitate the identification of an optimal medication for the patient. They associated these outcomes with reduced long-term cost-savings for the Canadian healthcare system.

*“I think it should be funded by the government, because I think there’s costs to the healthcare system for, if you’re on medications that aren’t working, there’s costs to the healthcare system—more frequent doctor’s visits, there may be socioeconomic costs, societal costs.”*

*“This could potentially be… the first step to recovery, which I think would, you know, limit some of the costs that people are already paying, that their tax money is already going towards.”*

An overall lack of literature showing the clinical efficacy of PGx testing, broadly, was cited as a key barrier to endorsing public coverage of testing. (Note that at the time of this focus group in October 2019, there was only one well-powered randomized controlled trial (RCT) in the literature [31], whereas currently, in 2023, there are seven RCTs published with an overall positive meta-analysis [5].

*“I’d want a larger cohort first. You know?... I’d want more data before making a judgment.”*

*“I agree with that…if there were some hard facts that came out, of success and positives, then I wouldn’t mind it being paid as a taxing thing because there’s so many people suffering from depression, and there’s so many people that can’t even afford, there’s some, there’s people on the street who are suffering from depression and it should just be accessible to everybody. So, I wouldn’t mind the masses, once it’s proven information, to be able to access this….But, once it’s accurate, you know.”*

### 3.3. PGx Results-Sharing and Accessibility

Privacy of test results and genetic data was flagged as an important consideration for clinical adoption across two groups. In particular, participants reached a consensus that they should have agency over whom in their care network may receive access to their test results. A suggested general comment was storing their genetic data on a secure platform that their healthcare providers could access. Participants agreed upon the benefits of communicating test results between their psychiatrist and general practitioner.

*“Ultimately, at the end of the day, it’s your results, your test, your genetics going into it. So, you should be able to have the right to make decisions on where you want to put that.”*

*“It would be nice to have a psychiatrist be able to communicate with a GP, and both of them have some ideas”*

Participants identified stakeholders with whom they would want to share and discuss test results; of these, general practitioners were most commonly identified. One participant explained that, in their view, general practitioners have the “most information” about the patient’s overall health and wellness.

*“I think that the control [over results-sharing] should be up to the patient.”*

Moreover, per the guidelines of this study, test results were directly sent to the patient’s physician to be later disclosed and explained to the patient by the physician. However, some patients had issues accessing their test results from their referring physician, which was in part due to a lack of communication between the physician and the patient. Another noteworthy mention from some patients was the lack of physical locations to receive PGx testing. ‘’Geography” was mentioned to be a barrier to receiving the test, where patients outside larger urban settings had a lack of access to PGx testing.

### 3.4. Clinical Utility (Generalizability)

Clinical utilization of PGx testing in psychiatry was assessed based on the experience of participants after taking the recommended medication. The GeneSight report categorized medications into green/yellow/red bins (the green bin indicates more likely recommended medications compared to the red/yellow bins). These recommendations were based on the likelihood of having fewer side effects and non-response.

*“I can honestly say it’s the first time I ever took a drug without side effects”*

*“I’ve been on antidepressants since 2004, and every antidepressant that I had been on up until then, except the one I’m currently on, was on the red list”*

The majority of participants had previously gone through a trial-and-error of multiple medications before the PGx test. However, the participants reported that the test results informed their decision in choosing the guided medication and which potential medications to avoid in the future. Moreover, one participant alluded to gaining “hope” by having access to the test results. This sentiment was highlighted by more participants, who felt the test results reinforced a sense of direction in terms of achieving remission. In other words, participants were more likely to adhere to their medication as prescribed.

*“I got the test, and when I looked at the range, the medications I was taking before were all in the red”*

*“I’m giving it all the time it’s going to take to get through the medication”*

### 3.5. Prospective Benefits

Consistent with the inclusion criteria for the IMPACT study, all participants had experienced failed medication trials, and the negative impact of these experiences was discussed across focus groups.

*“I tried several medications…and when I first tried them, I couldn’t even deal with them. I just said no. I stopped because of the side effects.”*

Benefits of the test were discussed, including those experienced by the participants and prospective benefits with regard to the test’s potential for clinical adoption and its potential to improve upon the current trial-and-error approach to medication prescription.

Two participants reported a successful medication trial after having their prescription altered to match the recommendation of the PGx test report.

*“I can honestly say it’s the first time I ever took a drug without side effects…it was awesome.”*

*“It helped me a lot with the dosage, like my results said that I had an ultrarapid metabolizer, so right now, the medication I’m on, I’m on the highest possible dose, and I was less hesitant to go to one of the higher doses because I know that I metabolize it faster.”*

Other participants reported that the PGx report increased their knowledge regarding avoiding the less recommended medications. As one participant mentioned, ‘’I got the test, and when I looked at the range, the medications I was taking before were all in the red”. In addition, many remained enthusiastic about PGx testing. Again, this optimism was largely due to their belief in its potential to allow others experiencing mental illness to avoid side effects and adverse events during medication trials. Several participants also shared that the PGx test provided them with a new understanding of their treatment journey and outcomes. Some also shared that the test gave them hope for a better treatment experience.

*“Even though it didn’t provide me with any information that could be useful, I would still do the test in a second…it just gives you a little bit more control in terms of knowing which meds are not considered the best for you and which ones could [be] potentially. Even the potential of having the medication show up on that list was good for me.”*

*“I think that it is gonna give a lot of people hope. I think I heard recently that it takes like seven medications on average before you find the right medication. So, this could potentially alleviate some of that.”*

*“Even though the research isn’t complete [and is] in its initial stages, it gives you a bit of hope and a bit of a compass…to start with, and maybe save me…from all that time where I don’t want to be.”*

Nevertheless, some participants cautioned that prospective users may be discouraged by results that do not include medications in the ‘Use as Directed’ category or in the event that medications listed in the ‘Use as Directed’ category do not lead to alleviation of mental illness symptoms. Indeed, some participants reported that they did not experience clinical improvements from medications that were listed in the ‘Use as Directed’ category and had been disappointed by this outcome. Again, improved physician education regarding the clinical opportunities and limitations of PGx testing and the subsequent plain-language communication of these opportunities and limitations to users was identified as a way to mitigate potential distress associated with test results.

### 3.6. IMPACT Experience

As the participants of this focus group were all recruited from the IMPACT study and had experienced PGx testing, the feasibility and experience of receiving the test were discussed. A core theme of the IMPACT experience was coded, including positive and negative sub-themes. All comments relevant to the patient’s experience from PGx testing were analyzed and coded as positive or negative.

A total of 39 references were coded as IMPACT experience, consisting of 36 positive and three negative comments, including opportunities for improvement. The negative comments were expressed by two participants, each of whom stated concern regarding the need for further development of physician and patient communication, and one participant cited a lack of sufficient physician knowledge on PGx testing (these concerns are discussed in greater detail in Section 3.1). Among positive experiences, participants found the overall process of the test to be smooth and easy to carry out. Furthermore, some participants mentioned that the test results reinforced their decision to take the correct medication, resulting in a sense of validation and guidance. As previously mentioned, one participant mentioned that the test results did not alleviate their concerns.

## 4. Discussion

The literature contains considerable discussion on PGx testing, with support for the potential efficacy of PGx in aiding in the successful selection of medication and reducing healthcare costs [32,33]. However, far fewer studies have been published examining public opinions regarding PGx testing. Given that the public is the consumer of this testing, their opinions are important to inform both benefits and areas for improvement. To contribute to the literature assessing stakeholder opinions of PGx testing, we conducted a focus group to evaluate the experiences of a group of individuals who have experienced using a PGx test. To the best of our knowledge, this study is unique in this regard.

This study revealed that most participants (91%) had a positive opinion of PGx testing but also provided comments on areas for improvement. The participants in this study shared notable barriers to their ability to benefit from PGx testing in a personally meaningful way, as well as corresponding needs that should be met in order to overcome these barriers. The lack of physician awareness and familiarity with PGx testing was considered the most significant barrier to receiving optimal benefits from the test. Other than direct-to-consumer testing, physicians regulate access to and dissemination of results of the PGx test used in our IMPACT study. For this physician-controlled method of test distribution to be fully effective, it is helpful for physicians to have a working knowledge of PGx testing. On a related note, participants across groups also stressed the need for improved education of the public and potential users of PGx tests regarding genomic medicine and PGx tests, generally. Thus, the results of this study call for a two-pronged knowledge mobilization approach. One approach would be targeted at physicians, and the second would be geared toward educating the general public. Accessible information regarding the potential benefits and existing limitations of research and innovation in personalized medicine, as well as an introduction to PGx tests and interpreting test results, could be particularly beneficial for each of these groups.

Participants also expressed a desire to have agency over sharing their test results, which highlights an important consideration related to data governance and sharing between direct-to-consumer testing vs. testing administered by a physician: the latter may ensure secure data storage via confidentiality policies to which healthcare staff and institutions are accountable. However, the former allows for more agency over sharing testing results, which may provide users with a greater sense of autonomy and empowerment. In this study, participants reported diverse experiences regarding their physicians’ willingness and ability to discuss their PGx test results; these experiences may influence their attitudes regarding data governance. While an analysis of the ethics of data governance and their implications for clinical adoption of PGx testing is outside the scope of this study, we encourage future research to explore this subject in greater detail, with a view toward establishing best practices for clinical adoption that address users’ desire for autonomy over their genetic data while also considering factors related to data security and storage.

Participants shared several benefits regarding their personal experience using a PGx test. For the two participants who experienced a successful medication trial following guidance from the PGx test, the test offered relief and renewed assurance in their treatment journey. Those who did not experience a successful medication trial following guidance from the PGx test shared a belief that PGx testing carries the potential to inspire hope in those who have experienced multiple unsuccessful medication trials. This is a testament to the need to (i) develop alternatives to trial-and-error medication prescription and (ii) reinforce community and healthcare supports to ease the hardship of the trial-and-error process. Continued research discoveries in the field of personalized medicine may support each of these needs.

The ideas and experiences reflected by the participants in this study correspond to 2019. Since then, various factors, such as the COVID-19 pandemic, might have altered the opinions of some participants. In addition, the sample size of overall participants in the focus groups was modest (*n* = 11), and the majority of participants were of European ancestry (*n* = 9). We encourage future studies collecting qualitative data regarding participants’ opinions and experiences of PGx testing to reflect a wider and more diverse range of opinions and experiences after PGx testing. Furthermore, although all facilitators received the same informal training and all participants were encouraged to provide their perspectives in response to each question, differences between group dynamics may have influenced participant responses.

Enrollment criteria for the focus group spanned urban, suburban, small-town, and rural communities. The focus group took place in a downtown urban setting, and this may have created biases in sampling, favoring urban participants. It is also important to note that other internal or external factors could have influenced the success of patient medication trials during the IMPACT study that we did not account for in the current study, such as age, gender, diet, and smoking, which may alter drug effectiveness. Patients may also have been impacted by the attitudes of their physician, who may have a personal preference for prescribing psychiatric medications based on familiarity and frequent usage instead of following the PGx test guidelines. It is also important to note that other internal or external factors could have influenced the success of patient medication trials during the IMPACT study that we did not account for in the current study, such as age, gender, diet, and smoking, which may alter drug effectiveness. Patients may also have been impacted by the attitudes of their physician, who may have a personal preference for prescribing psychiatric medications based on familiarity and frequent usage instead of following the PGx test guidelines. This study also did not account for participants’ overall level of education or employment status; these factors may have influenced participant attitudes toward clinical adoption, particularly regarding PGx test cost considerations (e.g., government subsidization of testing and willingness to pay out-of-pocket for PGx tests). Accounting for such factors in future research may yield additional insights regarding attitudes toward clinical adoption. It is possible that generalizability might have been limited since all participants received the same format of the PGx test (GeneSight Psychotropic (Myriad Genetics^TM^).

It should also be noted that evidence supporting the clinical efficacy of the PGx test used by participants, GeneSight Psychotropic (Myriad Genetics), has previously been reviewed by Ontario Health, the provincial oversight body regulating healthcare and health innovations in Ontario, Canada [34]. The review process reported concerns about the quality of evidence and economic cost-benefit supporting this test, precluding it as of 2021 from government subsidization in Ontario, Canada. At that time, the government did not have the results of this, or any other focus group, of patients who had taken the test. Also, as previously stated, in 2023, there are several more RCTs of PGx testing with significant positive results in the literature. In addition, PGx implementation in parallel fields, such as cancer research, has now been reported to have a positive impact on patient outcomes, with 76% of patients showing interest in learning more about PGx testing and 64% believing that PGx would significantly improve their cancer care [34].

## 5. Conclusions

Though PGx tests are commercially available in several countries, internationally, they have not yet been routinely adopted for clinical use in psychiatry. To add to our repertoire of research related to PGx testing, the current study aimed to understand patient opinions and needs related to PGx testing in psychiatry by conducting focus groups with patients who had used PGx testing during their psychiatric care. The overall outlook regarding PGx testing was positive, with patients highlighting that the test aided their medication selection, reduced hesitancy to make a medication change, and induced hope for future psychiatric care. The patients also demonstrated a willingness to pay for the test and the supportingsupport of coverage of testing coverage bythrough the use of taxpayer funds by the government. Key needs were also reported, including improved physician and public education regarding PGx testing and associated research, improved translation of test results for patients, and a need for more accessibility in remote areas and for those with lower incomes. Given the steady growth of PGx research and increasing interest among physicians and patients, the results of this study demonstrate opportunities for further patient-centred research regarding PGx testing and optimization of PGx use in psychiatric settings.

## Figures and Tables

**Table 1:** Principal Questions for the Focus Group.

1-How long did you participate in the study (IMPACT), and how would you describe your experience?
2-What are some benefits that you (personally) experienced from the test?
3-As a participant, did you have any concerns or difficulties with the test?
4-Please indicate the maximum amount you’d be willing to pay for this test out-of-pocket.
5-Would you support an increase in taxes for pharmacogenetic testing to be subsidized by the government?
6-Who needs to have access to the genetic test results for it to be the most effective for your care?
7-What could be some barriers to your ability to access a pharmacogenetic test?
8-If you oversaw making pharmacogenetic testing available to the public, what do you think would be the most important things to consider?

**Table 2:** Focus group participants’ characteristics (*n* = 11).

Characteristic	Participants
**Gender**	
Female	8
Male	3
**Age**	
18–29	3
30–49	3
50–64	4
65+	1
**Ancestry**	
European	9
East Asian	2

## Data Availability

The data used to support the findings of this study are available from the corresponding author upon reasonable request.

## Appendix A. Focus Group Questions

**1. Let’s start by discussing the IMPACT study. For how long did you participate in the study? What was the best part of your experience?**

- This question directly solicits feedback from participants re: their experience in the IMPACT study. From this question, we can have a better understanding of the overall experience of the testing process.

**2. What are some immediate and future benefits that you (personally) experienced or think you could experience from pharmacogenomic testing over a longer term?**

- From this question, we wish to learn about what, if anything, participants enjoyed about the process of being tested. This information will help us identify the patient need for pharmacogenomic testing.

**3. As a participant, did you have any concerns or difficulties with the test?**

- From this question, we wish to learn about any negative experiences the participants endured during the process of being tested. This information will help us identify potential areas for improvement/barriers to be navigated prior to implementation of pharmacogenomic testing.

**4. The maximum market price of this test could range from $800–1500. This is prohibitive for many people. Please indicate on the paper in front of you (everyone has one in front of them) the maximum amount you’d be willing to pay for this test out-of-pocket. When you’re done, put your paper in the box in front of me (i.e., the facilitator).**

- As the number of commercially available pharmacogenomic tests increases, patients can expect to see a diverse range of price points. This question directly relates to best practices for implementation of pharmacogenomic tests; with this information, we hope to get an estimate of the most realistic amount that target users of pharmacogenomic tests can afford to pay out-of-pocket. This information has significant implications for future policy (e.g., by government or insurance companies) regarding reimbursement for pharmacogenomic testing.

**5. There is the possibility that the government of Ontario could subsidize (or reimburse) part or all of this test. If it were to do so, it would have to use taxpayer money. Would you support an increase in taxes in order for this test to be subsidized by the government?**

- Consideration of a realistic but effective implementation strategy is an important aspect of translational research. The Canadian healthcare system is provincially regulated. We want patients to understand that, should they wish to have pharmacogenomic testing reimbursed by the government, this could result in increased taxes. With this information, we may better understand reimbursement from the patient’s point of view.

**6. Different patients have different kinds of clinicians and supporters in their treatment network. Who needs to have access to the genetic test results in order for it to be most effective for your care (i.e., PSWs, nurse practitioners, pharmacists, other caregivers?)**

- This question relates both to identification of the patient need and the development of an effective implementation strategy. We wish to better understand how results from pharmacogenomic testing may be best distributed to optimize patient benefit. This information has implications for policy development that includes everyone in a patient’s healthcare team.

**7. What could be some barriers to your ability to access a pharmacogenomic test?**

- Access to effective services is a key issue for patients seeking mental healthcare. This information will allow us to identify and understand some of these key issues to patients’ ability to access pharmacogenomic testing. We may then develop approaches to addressing these issues.

**8. If you were in charge of making this genetic test available to the public, what do you think would be the most important things to consider?**

- This question gives participants an opportunity to share any additional factors that they think are important to consider regarding implementation of pharmacogenomic testing. This information may contribute to recommendations to government and other key stakeholders for a patient-centred implementation strategy.
